# Bringing the Hospital to the Patient: First Treatment of Stroke Patients at the Emergency Site

**DOI:** 10.1371/journal.pone.0013758

**Published:** 2010-10-29

**Authors:** Silke Walter, Panagiotis Kostpopoulos, Anton Haass, Stefan Helwig, Isabel Keller, Tamara Licina, Thomas Schlechtriemen, Christian Roth, Panagiotis Papanagiotou, Anna Zimmer, Julio Vierra, Heiko Körner, Kathrin Schmidt, Marie-Sophie Romann, Maria Alexandrou, Umut Yilmaz, Iris Grunwald, Darius Kubulus, Martin Lesmeister, Stephan Ziegeler, Alexander Pattar, Martin Golinski, Yang Liu, Thomas Volk, Thomas Bertsch, Wolfgang Reith, Klaus Fassbender

**Affiliations:** 1 Department of Neurology, University Hospital of the Saarland, Homburg, Germany; 2 Zweckverband für Rettungsdienst and Feuerwehralarmierung, Saarland, Germany; 3 Department of Neuroradiology, University Hospital of the Saarland, Homburg, Germany; 4 Department of Anaesthesiology and Intensive Care, University Hospital of the Saarland, Homburg, Germany; 5 Department of Clinical Chemistry, Nürnberg Hospital, Nürnberg, Germany; Kenya Medical Research Institute, Kenya

## Abstract

**Background:**

Early treatment with rt-PA is critical for favorable outcome of acute stroke. However, only a very small proportion of stroke patients receive this treatment, as most arrive at hospital too late to be eligible for rt-PA therapy.

**Methods and Findings:**

We developed a “Mobile Stroke Unit”, consisting of an ambulance equipped with computed tomography, a point-of-care laboratory system for complete stroke laboratory work-up, and telemedicine capabilities for contact with hospital experts, to achieve delivery of etiology-specific and guideline-adherent stroke treatment at the site of the emergency, well before arrival at the hospital. In a departure from current practice, stroke patients could be differentially treated according to their ischemic or hemorrhagic etiology even in the prehospital phase of stroke management. Immediate diagnosis of cerebral ischemia and exclusion of thrombolysis contraindications enabled us to perform prehospital rt-PA thrombolysis as bridging to later intra-arterial recanalization in one patient. In a complementary patient with cerebral hemorrhage, prehospital diagnosis allowed immediate initiation of hemorrhage-specific blood pressure management and telemedicine consultation regarding surgery. Call-to-therapy-decision times were 35 minutes.

**Conclusion:**

This preliminary study proves the feasibility of guideline-adherent, etiology-specific and causal treatment of acute stroke directly at the emergency site.

## Introduction

Stroke is the main cause of chronic disability in adults and a major cause of death and dementia [1], [2]. Recanalization of occluded arteries by systemic thrombolysis with recombinant tissue plasminogen activator (rt-PA) within 3 hours after onset of ischemic stroke significantly reduces disability and death [3]–[5]. However, before administration of rt-PA therapy, neurological examination, imaging, and laboratory analyses are required to exclude hemorrhagic stroke, stroke mimics or other contraindications for thrombolysis [4]–[7]. Consequently, implementation of rt-PA therapy within the narrow therapeutic window is difficult to achieve in clinical routine. Despite the potential for thrombolytic therapy to improve the outcomes of patients after ischemic stroke, only 15–40% arrive at the hospital early enough to be eligible for treatment [8], [9]. Thus, studies have estimated that only 1.8%–3.0% of all ischemic stroke patients in the United States are treated with rt-PA [10], [11].

Acute stroke management needs to be reconfigured to allow rapid screening and treatment of stroke patients for time-limited therapy. To accomplish this, we realized a specialized ambulance, Mobile Stoke Unit (MSU, “Stroke-Ambulance”) for prehospital stroke treatment that provides all diagnostic tools and stroke medicine competence needed for therapeutic decisions directly at the site of the emergency as hypothesized before [12]. Here, we present first patients demonstrating that guideline-adherent and etiology-specific treatment of ischemic and of hemorrhagic stroke, as early as the prehospital phase of stroke management is feasible in clinical reality.

## Results

### Patient with prehospitally treated ischemic stroke

**Figure 1:**
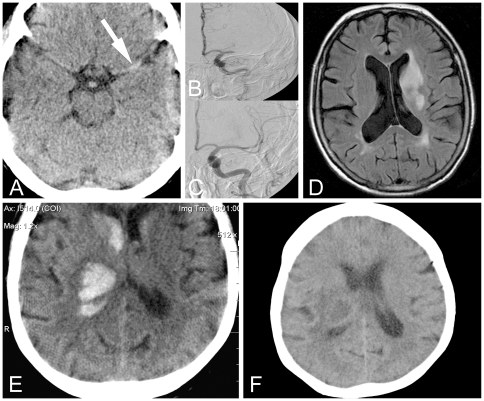
Brain imaging in prehospitally treated stroke patients . Patient with ischemic stroke: Prehospital CT excluded contraindications for thrombolysis as a precondition for prehospital rt-PA thrombolysis (A). The “hyperdense middle cerebral artery” sign (arrow) suggested middle cerebral artery occlusion that was later confirmed in hospital by angiography (B), and reopened by intraarterial recanaliziation (C). Diffusion-weighted magnetic resonance imaging at day 7 showed the residual infarction (D). Patient with hemorrhagic stroke: Prehospital CT scan allowed immediate diagnosis of intracerebral hemorrhage with ventricular extension (E), as a precondition for prehospital differential blood pressure management and telemedicine consultation with hospital experts. CT performed at day 24 shows the residual lesion (F).

A 66-year-old right-handed woman (case 23 of the current MSU program and the first patient with thrombolysis bridging therapy) suddenly collapsed. In the prehospital neurological examination, the patient was awake, exhibiting a fixed ocular deviation to the left, a very severe dysarthria, a severe right-facial paresis and a paresis of the right upper (grade 0-1/5) and lower (grade 2/5) extremities (NIHSS: 16, Rankin: 5, Barthel: 0). Prehospital point-of-care-based laboratory analysis values are shown at table 1. The prehospital CT excluded a hemorrhage, demarcated infarction or other pathological findings, but showed a left “hyperdense middle cerebral artery”-sign (Fig. 1A, arrow), indicative of a vessel occlusion [13]. Prehospitally, acute cerebral ischemia was diagnosed. The stroke physician contacted hospital neurologists and neuroradiologists to arrange bridging [14] to later intra-arterial recanalization. Based on the patient's weight of 70 kg, the total rt-PA dosage (0.9 mg/kg body weight) was determined to be 63 mg [3], [7]. Prehospital IV-thrombolysis was started with a 6 mg rt-PA bolus, followed by infusion of further 47 mg, with plans to save 10 mg for later intra-arterial thrombolysis, if necessary. Systolic blood pressure was closely monitored. After IV thrombolysis, the dysarthria and paresis of the lower extremity (grade 2-3/5) slightly improved.

**Table 1:** Point-of-care-laboratory examination in patients with prehospital stroke treatment.

Laboratory test	Unit	Patient 1	Patient 2
leukocytes	10^3^/µl	8.0	5.3
platelets	10^3^/µl	280	134
erythrocytes	10^6^/µl	5.36	4.19
hemoglobin	g/dl	16.0	13.4
hematocrit	%	47	42
INR		1.3	1.2
aPTT	seconds	46*	32
p-amylase	U/ml	23	160
γ-glutamyltransferase	U/ml	25.1	92.0
glucose	mg%	99	75

*normal aPTT values for Hemochron Jr. ITC Edison, USA below 42 seconds.

At the hospital, a CT angiography and a digital subtraction angiography confirmed an obstruction in the proximal segment of the left middle cerebral artery (Fig. 1B) that was reopened by consecutive mechanical recanalization [15] and intra-arterial thrombolysis (Fig. 1C). The neurological symptoms markedly improved until discharge at day 9 to an only slight dysarthria, a minor facial paresis (NIHSS: 2; Rankin: 1; Barthel: 80). The MRI at day 7 showed the residual basal ganglia infarction at the left hemisphere (Fig. 1D). A cardioembolic etiology was diagnosed based on the detection of a previously unknown atrial fibrillation.

### Patient with prehospitally treated hemorrhagic stroke

A 63-year-old right-handed woman (case 2 of the program, first case of cerebral hemorrhage) suddenly developed slurred speech, drooling and left hemiparesis. The prehospital neurological status by the stroke physician revealed a severe dysarthria, a left-sided facial paresis and a paresis of the left upper (grade 2/5) and lower (grade 3/5) extremities (NIHSS 7, Rankin 4, Barthel 50). Further history by relatives revealed presence of hypertension. Complete prehospital point-of-care-based laboratory analysis is shown at table 1. Prehospital CT revealed an extensive intracerebral hemorrhage in the right basal ganglia with rupture into the ventricles (Fig. 1E). Systolic hypertension of 200 mmHg was prehospitally adjusted with IV urapidil to 150 mmHg and monitored every 5 minutes. CT scans were transmitted to the hospital to inform neuroradiologists and neurosurgeons, thereby accelerating a decision against need of acute surgical interventions (e.g., ventricular catheter).

In the hospital, the patient markedly improved and was discharged at home at day 38 with a residual mild left-sided facial paresis and latent paresis of the left upper (grade 5-/5) and lower (grade 5-/5) extremities (NIHSS: 3, Rankin: 3, Barthel: 60). Follow-up CT at day 24 shows the residual lesion (Fig. 1F). Based on the history of hypertension and the further work-up a hypertensive intracerebral hemorrhage was diagnosed.

### Response times

For patient 1, an overall call-to-therapy decision time of 35 minutes was achieved. Specifically, the symptom-onset-to-call time was 25 minutes; the call-to-MSU-arrival time, 20 minutes (distance of 12 km); the call-to-door time, 65 minutes; the MSU-arrival-to-door time, 45 minutes; the call-to-neurological examination time, 25 minutes; the call-to-laboratory examination time, 34 minutes; and the call-to-CT time, 35 minutes.

For patient 2, an overall call-to-therapy decision time of 33 minutes was reached. The symptom-onset-to-call time was 17 minutes; the call-to-MSU-arrival time, 11 minutes (distance of 4.5 km); the call-to-door time, 63 minutes; the MSU-arrival-to-door time, 52 minutes, the call-to-neurological examination time, 18 minutes; the call-to-laboratory examination time, 31 minutes; and the call-to-CT time, 33 minutes.

## Methods

In the context of a long-term monocentric randomised clinical trial (ClinicalTrial.gov.Identifier: NCT00792220) started in 2009 with an expected end not before 2013, we analyzed the feasibility in clinical practice of a unique healthcare-delivery system for provision of prehospital treatment to acute stroke patients. The MSU program has been integrated into the emergency medical chain in a mixed urban and rural setting 20 km around the University hospital, Homburg, Saarland, Germany. Our hospital stroke service admits patients from a core region with a radius of approx. 16 km (150.000 inhabitants), although this major stroke service treats, in addition, patients beyond this core region on an irregular basis. In 2009, 804 stroke patients were admitted; of these 10.5% obtained thrombolysis, with call-to-door time of 44±14 minutes, ambulance-arrival-to-door time of 37±13 minutes, followed by an intrahospital door-to-needle time of 59±37 minutes.

The emergency medical system dispatch centre screens potential stroke cases using a modified Rosier questionnaire [16], and when appropriate, dispatches the MSU together with the standard emergency support system, which in Germany includes an emergency physician for critically ill cases. The MSU program was approved by the Regional Ethics Committee of the “Medical Association” of the Saarland, Germany. The patients described in this manuscript have given written informed consent to publication of their case details.

**Figure 2:**
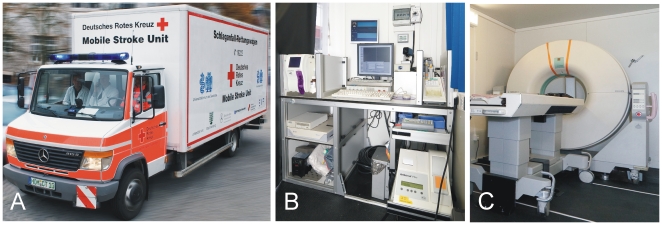
Mobile Stroke Unit. An ambulance (A) equipped with point-of care laboratory system and telemedicine devices (B) and CT (C) required for prehospital stroke treatment.

The MSU system includes a paramedic, a physician trained in stroke medicine, and a neuroradiologist (optional for this pilot phase, as there is also telemedicine contact to a hospital neuroradiologist). The MSU (Fig. 2A-C) is an ambulance (Mercedes-Benz Vario 815D) that includes, apart from the conventional ambulance equipment, an accumulator-driven and lead-shielded CT (Tomoscan M, Philips), a telemedicine system (Meytec Inc. Werneuchen, Germany) enabling transmission of “digital imaging and communication” data from CT scans or real-time video of clinical examination of patients via UMTS, i.e., high speed downlink packet access or alternative network standards to the “picture archiving and communication system” of the hospital, and a point-of-care laboratory system. With the latter, platelet count, leukocyte count, erythrocyte count, hemoglobin and hematocrit (PocH 100i, Sysmex, Hamburg, Germany), international normalized ratio and activated partial thromboplastin time (Hemochron Jr., ITC, Edison, NY, USA) and γ-glutamyltransferase, p-amylase and glucose (Reflotron plus, Roche Diagnostics Mannheim, Germany) are quantified as requested by current stroke management guidelines [4], [5], [7].

## Discussion

Although IV thrombolysis with rt-PA, initiated within 3 hours after the onset of symptoms, is the only medical therapy currently approved for acute ischemic stroke [4], [5] this therapy is disappointingly underused [8], [9]. Here we report etiology-specific and causal treatment of acute stroke, i.e., cerebral ischemia and cerebral hemorrhage, directly at the site of the emergency rather than waiting until hospital arrival. This was accomplished by a newly developed strategy to deliver time-dependent treatment at the prehospital phase of stroke management, using an ambulance with integrated CT, rapid point-of-care laboratory system and telemedicine capabilities.

The two reported complementary stroke cases, one with cerebral ischemia, the other with cerebral hemorrhage illustrate the broad range of medical solutions made available by use of the MSU. The MSU is not limited to the delivery of prehospital thrombolysis; as the name implies the MSU encompasses all major aspects of prehospital stroke medicine such as prehospital organization of bridging to intra-arterial recanalization [14], prehospital inquiry regarding the need for surgical or other intervention with hospital experts via telemedicine, guideline-adherent and etiology-specific prehospital management of physiological variables (i.e., blood pressure), and prehospital decision-making about the target hospital (e.g., a more distant hospital with stroke unit, neurosurgery or neuroradiology vs. a closer hospital without those resources).

The immediate diagnosis of intracerebral hemorrhage in patient 2 enabled for the first time guideline-adherent differential blood pressure management [17], [18] within the prehospital stage of stroke management. Guidelines on blood pressure are different for ischemia (tolerating systolic values up to 185–220 mm Hg to enhance cerebral perfusion pressure) [4], [5] compared to hemorrhage (intervening at systolic values of 160–180 mm Hg to avoid ongoing bleeding [17], [18], although there is, at present, less evidence for clinical relevance of hyperacute blood pressure reduction in hemorrhagic stroke than for hyperacute thrombolysis in ischemic stroke.

Strong pathophysiological and clinical evidence suggests that time gain translates to better clinical outcome in treatment of stroke (“time is brain” concept) [19]–[20]. In each of the two cases, call-to-therapy-decision times were approximately 35 minutes, and clinical outcomes were good. These times dramatically break current time limits for stroke management, i.e., the door-to-therapy-decision times of 60 minutes defined as a goal by current guidelines [4], [5] or the >60-minute times encountered in daily clinical practice [21]. The marked time gains reported here resulted not only from reduced times spent in transport or diagnostic work-ups, but also from increased efficiency in crucial interfaces between paramedics, emergency physicians, neurologists, neuroradiologists, neurosurgeons, or laboratory personnel. If the patient is not in a critical condition, as judged by the physician, a potential delay in the time to hospital arrival should no longer be crucial once the time-dependent, guideline-adherent diagnostic work-up and therapy have already been delivered in the field.

Concerning the point-of-care laboratory system, we performed earlier investigations showing generally good agreement between values derived from a point-of-care laboratory and from a centralized hospital laboratory, although room for some technical improvement exists, i.e., of aPTT and INR. From a technical point of view, we observed that the various components of the MSU functioned as expected, although we initially encountered technical problems with the CT at extreme temperatures.

Treating acute stroke immediately at the site where the patient is found has the potential to prevent brain damage, thereby reducing individual suffering and costs for stroke care for years and decades. In the future, such MSU can be miniaturized, i.e., using a smaller CT already commercially available, and could include future diagnostic and therapeutic tools (e.g., biomarkers, sonothrombolysis, neuroprotectives or hemorrhage treatments, if proven to be relevant [22]). Though large randomized multi-center studies are needed to conclusively establish the benefit and to determine the optimal setting (rural vs. urban), the optimal integration in the regional emergency chain (e.g., dispatching the MSU ambulance alone rather than combined with the conventional ambulance in selected cases), and the cost effectiveness of this MSU concept, this preliminary report demonstrates for the first time that delivery of guideline-adherent and etiology-specific treatment in the prehospital phase of stroke is feasible in clinical practice.
