# Correction: Bringing the Hospital to the Patient: First Treatment of Stroke Patients at the Emergency Site

**DOI:** 10.1371/annotation/f4013549-fbb8-4dbc-9757-73e6621c81b0

**Published:** 2011-03-08

**Authors:** Silke Walter, Panagiotis Kostpopoulos, Anton Haass, Stefan Helwig, Isabel Keller, Tamara Licina, Thomas Schlechtriemen, Christian Roth, Panagiotis Papanagiotou, Anna Zimmer, Julio Vierra, Heiko Körner, Kathrin Schmidt, Marie-Sophie Romann, Maria Alexandrou, Umut Yilmaz, Iris Grunwald, Darius Kubulus, Martin Lesmeister, Stephan Ziegeler, Alexander Pattar, Martin Golinski, Yang Liu, Thomas Volk, Thomas Bertsch, Wolfgang Reith, Klaus Fassbender

The 11th author's name was entered incorrectly. The correct name is: Julio Viera 

